# Bevacizumab Increases Endothelin-1 Production via Forkhead Box Protein O1 in Human Glomerular Microvascular Endothelial Cells In Vitro

**DOI:** 10.1155/2021/8381115

**Published:** 2021-12-06

**Authors:** Satoru Nihei, Junichi Asaka, Hiroaki Takahashi, Kenzo Kudo

**Affiliations:** ^1^Department of Pharmacy, Iwate Medical University Hospital, 2-1-1 Idaidori, Yahaba-cho, Iwate 028-3695, Japan; ^2^Division of Clinical Pharmaceutics and Pharmacy Practice, Department of Clinical Pharmacy, School of Pharmacy, Iwate Medical University, Iwate Medical University, 1-1-1 Idaidori, Yahaba-cho, Shiwa-gun, Iwate 028-3694, Japan

## Abstract

Molecular mechanisms underlying the nephrotoxicity associated with bevacizumab are unclear. Endothelin-1 (ET-1) is involved in podocyte injury and proteinuria, and its level increases in most cases of kidney disorders. Forkhead box protein O1 (FoxO1), a transcription factor, is a major determinant of ET-1 promoter activation and is regulated by protein kinase B (Akt) phosphorylation-dependent nuclear exclusion. We evaluated the effect of bevacizumab on ET-1 production in human glomerular microvascular endothelial cells (hGECs). We analyzed the changes in the mRNA and protein levels of ET-1 in hGECs treated with bevacizumab using real-time reverse transcription-polymerase chain reaction and enzyme-linked immunosorbent assay. Changes in the protein levels and phosphorylation status of Akt and FoxO1 in hGECs treated with bevacizumab were analyzed by western blotting. After cell lysis, FoxO1 protein was isolated from the cytoplasmic and nuclear fractions. We also investigated the effects of AS1842856 (a FoxO1 inhibitor) on bevacizumab-induced ET-1 production. Bevacizumab significantly and dose-dependently increased the mRNA and protein levels of ET-1 in hGECs (*p* < 0.05). Bevacizumab treatment also led to a decrease in phosphorylated Akt protein levels. Inhibition of Akt activity by LY294002 promoted ET-1 production. Bevacizumab also induced an increase in FoxO1 protein levels in the nucleus. Inhibition of FoxO1 activity by AS1842856 resulted in decreased ET-1 levels in bevacizumab-treated hGECs. ET-1 axis activation, Akt inactivation, and FoxO1 nuclear localization are the molecular mechanisms underlying bevacizumab-induced nephrotoxicity. Therefore, inhibition of renal ET-1 production could be a promising approach to protect against or treat bevacizumab-induced nephrotoxicity.

## 1. Introduction

Bevacizumab is a humanized monoclonal antibody targeting human vascular endothelial growth factor A (VEGFA) and is currently the most commonly used angiogenesis inhibitor for cancer treatment [1–3]. The effects of bevacizumab, such as antiangiogenesis and tumor suppression, are associated with the inhibition of VEGFA signaling [4, 5]. However, bevacizumab treatment is also associated with nephrotoxicity, including hypertension, proteinuria, nephrotic syndrome, and renal-limited thrombotic microangiopathy [6]. Bevacizumab-induced nephrotoxicity, characterized by major histological changes observed in glomerular disorders, results in severe defects in the glomerular filtration barrier that prevents the leakage of serum proteins into the urine [7–11]. Nevertheless, the exact mechanisms underlying bevacizumab nephrotoxicity are unclear.

The glomerular filtration barrier has three major components: (1) an internal layer of endothelial cells, (2) a middle acellular layer of the glomerular basement membrane, and (3) an external layer of epithelial cells, known as podocytes. Endothelial cells secrete various vasoactive agents, including vasodilatory nitric oxide, prostacyclin, vasoconstrictor endothelin-1 (ET-1), and thromboxane [12]. Under disease conditions, ET-1 level increases in various renal disorders, including diabetic nephropathy, glomerulonephritis, and preeclampsia [13, 14]. Increased circulating ET-1 levels are found in animals and patients treated with sunitinib, a multitarget VEGF receptor (VEGFR) inhibitor [15]. Inhibitors of the VEGF signaling pathway probably promote podocyte actin cytoskeleton disruption by promoting ET-1 production, resulting in heavy proteinuria [16–18]. However, whether bevacizumab increases ET-1 production in the vascular endothelium and the molecular mechanisms underlying bevacizumab-induced nephrotoxicity remains unclear. A recent report indicated that forkhead box protein O1 (FoxO1) transcription factor is a major determinant of ET-1 promoter activation [19]. In addition, the FoxO1 transcription factor is regulated by protein kinase B (also called Akt) phosphorylation-dependent nuclear exclusion [20].

In this study, we aimed to evaluate the effect of bevacizumab on ET-1 production in human glomerular microvascular endothelial cells (hGECs). The changes in mRNA and protein levels of ET-1 and the protein levels and phosphorylation status of Akt and FoxO1 in hGECs treated with bevacizumab were analyzed. Our study indicated that the “Akt/FoxO1” pathway could be involved in increased ET-1 production caused by bevacizumab. Deciphering this mechanism may help in understanding bevacizumab-induced nephrotoxicity.

## 2. Materials and Methods

### 2.1. Cell Culture

The hGEC primary cultures were purchased from CellSystems, and 4 to 6 passages were performed for all experiments. The cells were plated either in 24-well plates at a density of 1.0 × 10^5^ cells/well or in 60 mm culture dishes at a density of 1.0 × 10^6^ cells/dish and cultured until they reached 90% confluence. The cells were cultured in endothelial growth medium (EGM-2MV; Lonza, Basel, Switzerland) supplemented with 5% fetal bovine serum, 0.1% VEGF, 0.1% human epidermal growth factor, 0.1% R3insulin-like growth factor-1, 0.4% human fibroblast growth factor, 0.1% ascorbic acid, 0.04% hydrocortisone, 0.1% heparin, and 0.1% gentamicin/amphotericin B, according to the manufacturer's instructions. The cells were maintained at a humidified incubator at 37°C with 5% CO_2_. Monolayers of cells that were 90% confluent were serum-starved for 24 h before the experiments were performed. The cells were treated with 0.1 or 1 *µ*M bevacizumab. The rationale for the dose setting of bevacizumab in this study reflects the use of the drug in clinical practice [21]. The cells were also incubated with LY294002, a phosphatidylinositol-3 kinase (PI3K)/Akt pathway inhibitor, and AS1842856, a FoxO1 inhibitor, to determine the effect of Akt and FoxO1 inhibition on ET-1 production. The rationale for the dose setting of LY294002 and AS1842856 in this study reflects the minimum concentration needed to inhibit Akt and FoxO1 (data not shown).

### 2.2. Reagents

Bevacizumab was purchased from Chugai Pharmaceutical Co. (Tokyo, Japan). LY294002 and VEGFA were purchased from FUJIFILM Wako Pure Chemical Corporation (Osaka, Japan). AS1842856 was purchased from Calbiochem (Merck Millipore, Billerica, MA, USA). Antibodies against phospho-Akt (S473, Cat. No. ab81283), Akt (Cat. No. ab179463), phospho-FoxO1 (S256, Cat No. ab47326), FoxO1 (Cat. No. ab39670), and lamin-B1 (Cat No. ab133741) were purchased from Abcam (Cambridge, UK). Antibodies against glyceraldehyde 3-phosphate dehydrogenase (GAPDH) (Cat. No. sc32233) and mouse (Cat. No. sc2005) and rabbit (Cat. No. sc2004) immunoglobulin G (IgG)-horseradish peroxidase (HRP) conjugates were purchased from Santa Cruz Biotechnology, Inc. (Santa Cruz, CA, USA).

### 2.3. Real-Time Reverse Transcription-Polymerase Chain Reaction (RT-PCR)

Total RNA was isolated from samples using the RNeasy Mini Kit (Qiagen Company, Hilden, Germany). A total 2 *µ*g of RNA was transcripted into complementary DNA (cDNA) using the High-Capacity RNA-to-cDNA Kit (Applied Biosystems, Foster City, CA, USA). Quantitative PCR was performed using 7500 Real-Time PCR System (Applied Biosystems), TaqMan Universal Master Mix II (Applied Biosystems), TaqMan Gene Expression Assays (Applied Biosystems), and 200 ng of cDNA (corresponding to the amount of input RNA) from respective samples. Taqman assays utilized in this study were as follows: *ET-1*: Hs00174961_m1 and *GAPDH*: Hs02786624_g1. The relative level of *ET-1* mRNA was calculated by the 2−ΔΔCt method with data normalized to the *GAPDH* housekeeping gene [22]. The fold change in expression with respect to control (unstimulated cells) was calculated for all samples. The experiment was carried out in duplicate and repeated three times.

### 2.4. Western Blot Analysis

The cells were harvested and washed with cold phosphate-buffered saline. Total proteins were extracted from the samples using radioimmunoprecipitation assay buffer (FUJIFILM Wako Pure Chemical Corporation) containing protease and phosphatase inhibitor cocktails (Thermo Fisher Scientific, Inc., Rockford, IL, USA). Alternatively, nuclear and cytoplasmic protein extracts were isolated using the Nuclear and Cytoplasmic Extractor Kit (FUJIFILM Wako Pure Chemical Corporation) following the manufacturer's instructions. Insoluble materials were removed by centrifugation at 20,000 ×*g* for 10 min at 4°C. The supernatants were collected, and protein concentration was quantified using the bicinchoninic acid kit (Thermo Fisher Scientific, Inc.) following the manufacturer's instructions. An aliquot of 20 *μ*g of protein was loaded and separated on 7.5% and 12% SDS-polyacrylamide gel. After separation, the proteins were transferred onto polyvinylidene fluoride membranes. The transferred membranes were blocked with 5% nonfat milk in Tris-buffered saline containing 0.1% Tween-20 (TBST) for 1 h and incubated at 4°C overnight with the following primary antibodies: anti-p-FoxO1 (1 : 1000), anti-FoxO1 (1 : 1000), anti-GAPDH (1 : 5000), anti-lamin-B1 (1 : 1000), anti-p-Akt (1 : 1000), and anti-Akt (1 : 000). After incubation, the membranes were washed with TBST three times and incubated with HRP-conjugated anti-mouse and anti-rabbit IgG secondary antibodies (1 : 5000) for 2 h at room temperature. The membranes were washed with TBST five times and developed using the electrochemiluminescence reagent (Thermo Fisher Scientific, Inc.). The density of the products was quantified using ImageJ software. The experiment was carried out in triplicate and repeated three times.

### 2.5. Enzyme-Linked Immunosorbent Assay (ELISA)

The concentration of ET-1 in the supernatant was determined by sandwich enzyme-linked immunosorbent assay (ELISA) using a human ET-1 Quantikine ELISA kit (R&D Systems, Abingdon, UK). The ELISA kit was used following the manufacturer's instructions. ET-1 concentration in the aliquots of supernatants obtained from incubated hGECs was measured in duplicate. The results were analyzed using an SH-1200 microplate reader (Corona Electric Co. Ltd, Ibaraki, Japan) at a wavelength of 450 nm. According to the manufacturer, the sensitivity of the assay was 0.207 pg/mL. The results were calculated based on the standard curve and expressed in pg/mL. ET-1 protein concentrations were normalized for the cell number. The experiment was carried out in duplicate and repeated three times.

### 2.6. Statistical Analysis

Statistical analysis was performed using IBM SPSS. Results are representative of at least three independent experiments performed in triplicate and are expressed as mean ± standard deviation. Data are expressed as mean ± standard deviation. Analysis of variance was performed, followed by Tukey's multiple comparison test where appropriate to compare different groups. A *p* value of <0.05 was considered statistically significant.

## 3. Results

### 3.1. Bevacizumab Induced an Increase in ET-1 Production

**Figure 1:**
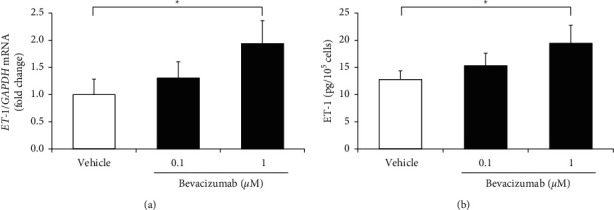
Bevacizumab induced ET-1 production in cultured human glomerular microvascular endothelial cells (hGECs). hGECs were plated in 24-well plates (1 × 10^5^ cells/well) for ELISA or 60 mm culture dishes (1 × 10^6^ cells/dish) for RT-PCR. hGECs that were 90% confluent were serum-starved for 24 h before the experiments were performed. hGECs were treated with 0.1 or 1 *µ*M bevacizumab for 8 h, RNA was extracted from the cells, and *ET-1* mRNA level was examined by real-time RT-PCR (a). The medium was collected and ET-1 protein level was examined by the ELISA (b). Data shown are means ± SD (*n* = 3; ^*∗*^*p* < 0.05). The experiments were repeated at least three times, with reproducible results.

We evaluated the effect of bevacizumab on ET-1 production in hGECs. First, to determine the *ET-1* mRNA level in bevacizumab-treated hGECs, we performed real-time RT-PCR. Compared with the control group, bevacizumab treatment significantly increased *ET-1* mRNA level in a dose-dependent manner; 0.1 *µ*M bevacizumab increased *ET-1* mRNA level by ∼31%, whereas 1 *µ*M bevacizumab increased it by ∼94% (Figure 1(a)). The medium was then collected and ELISA determined the ET-1 protein level. Bevacizumab treatment increased the ET-1 protein level in a dose-dependent manner compared to the control group; 0.1 mM bevacizumab increased the ET-1 protein level by ∼20%, whereas 1 *µ*M increased it by ∼52% (Figure 1(b)). Together, these results indicate that bevacizumab induces an increase in ET-1 synthesis and secretion in hGECs.

### 3.2. Bevacizumab Inactivates Akt Pathway

**Figure 2:**
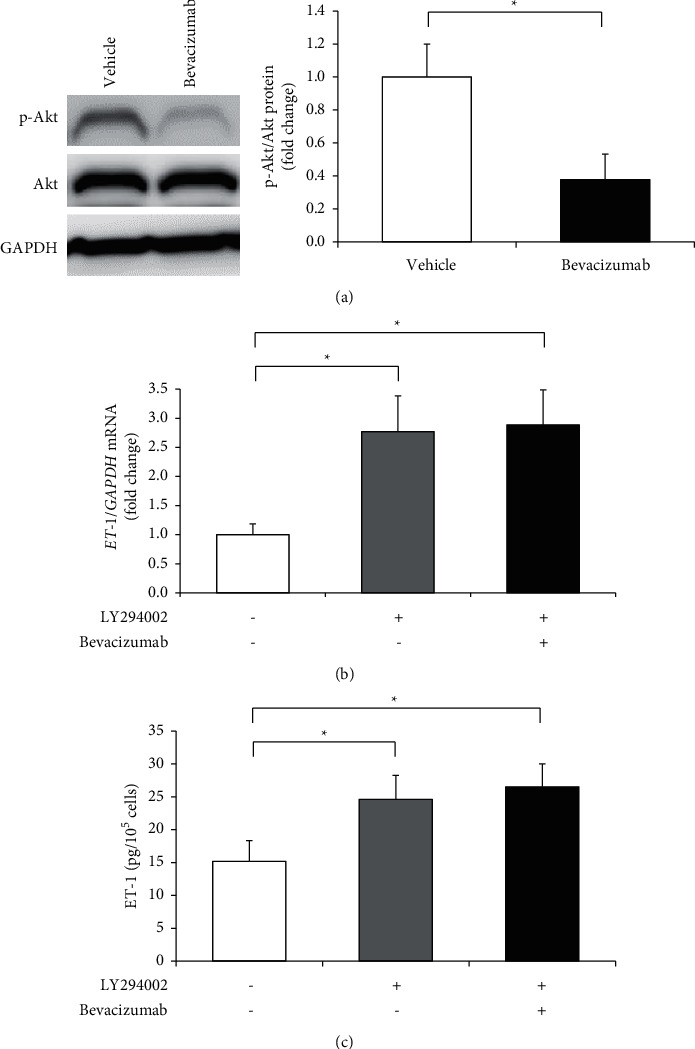
The Akt pathway was inactivated in human glomerular microvascular endothelial cells (hGECs) treated with bevacizumab. hGECs were plated in 24-well plates (1 × 10^5^ cells/well) for ELISA or 60 mm culture dishes (1 × 10^6^ cells/dish) for RT-PCR and western blot. hGECs that were 90% confluent were serum-starved for 24 h before the experiments were performed. hGECs were treated with 1 *µ*M bevacizumab or 10 *µ*M LY294002 (PI3K/Akt inhibitor) for 8 h, and western blots were used to evaluate the changes in Akt phosphorylation after bevacizumab treatment (a). The levels of *ET-1* mRNA (b) and ET-1 protein (c) were measured using real-time RT-PCR and the ELISA after LY294002 treatment. Data shown are means ± SD (*n* = 3; ^*∗*^*p* < 0.05). The experiments were repeated at least three times, with reproducible results.

**Figure 3:**
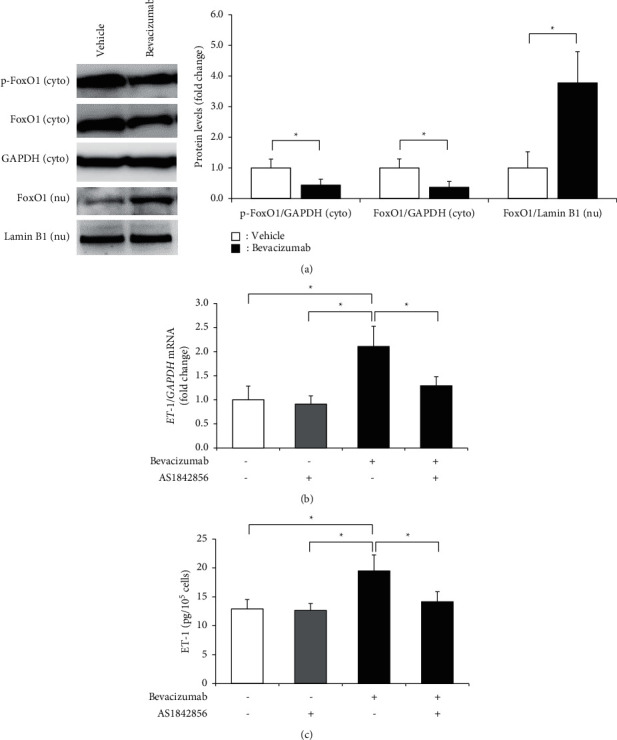
The nuclear localization of FoxO1 was increased in human glomerular microvascular endothelial cells (hGECs) treated with bevacizumab. hGECs were plated in 24-well plates (1 × 10^5^ cells/well) for ELISA or 60 mm culture dishes (1 × 10^6^ cells/dish) for RT-PCR and western blot. hGECs that were 90% confluent were serum-starved for 24 h before the experiments were performed. hGECs were treated with 1 *µ*M bevacizumab and 0.1 *µ*M AS1842856 (FoxO1 inhibitor) for 8 h, and western blots were used to evaluate the changes in FoxO1 protein level in cytosolic (cyto) and nuclear (nu) fractions after bevacizumab treatment (a). The levels of *ET-1* mRNA (b) and ET-1 protein (c) were measured using real-time RT-PCR and the ELISA after bevacizumab and AS1842856 treatment. Data shown are means ± SD (*n* = 3; ^*∗*^*p* < 0.05). The experiments were repeated at least three times, with reproducible results.

The Akt pathway—a classical signaling pathway responsible for prototypical endothelial functions, including the regulation of vascular tone, angiogenesis, and control of adhesion—may lead to a decrease in ET-1 production. Therefore, we determined whether bevacizumab affects Akt activity in hGECs. Western blotting showed that Akt was inactivated in hGECs after bevacizumab treatment, as indicated by a reduced phosphorylation status of Akt (Figure 2(a)). These results showed that the Akt pathway is a downstream target of bevacizumab. hGECs were incubated with LY294002, a PI3K/Akt inhibitor, to understand whether Akt inactivation contributed to the increased ET-1 production. As shown in Figure 3, compared with the control group, the LY294002 treatment significantly increased the mRNA and protein levels of ET-1 (Figures 2(b) and 2(c)). In addition, the combination of LY294002 and bevacizumab did not have an additional effect on ET-1 production; thus, Akt inactivation plays a role in increased ET-1 production caused by bevacizumab treatment.

### 3.3. Bevacizumab Induced an Increase in Nuclear Localization of FoxO1

FoxO1 activates the promoter of the *ET-1* gene. FoxO1 translocates from the nucleus to the cytoplasm by phosphorylation via upstream kinases, including Akt, which results in the loss of nuclear localization and transcriptional activity [20]. We performed western blotting to determine the FoxO1 protein level in the cytosolic and nuclear fractions of bevacizumab-treated hGECs. The FoxO1 protein level in the nuclear fraction was significantly increased in bevacizumab-treated hGECs (Figure 3(a)). Thus, bevacizumab is likely to inhibit Akt activity that promotes FoxO1 translocation from the nucleus to the cytoplasm, resulting in the increased nuclear localization of FoxO1. As nuclear FoxO1 localization was increased in bevacizumab-treated hGECs, we next investigated whether FoxO1 played a critical role in the bevacizumab-induced increase in ET-1 production. Bevacizumab remarkably increased the mRNA and protein levels of ET-1, which was alleviated by AS1842856 (Figures 3(b) and 3(c)). Thus, one mechanism by which bevacizumab increases ET-1 production is likely to involve FoxO1 phosphorylation by Akt. In summary, FoxO1 repression could abate a bevacizumab-induced increase in ET-1 production.

### 3.4. VEGFA Is Associated with the Suppression of ET-1 Production

**Figure 4:**
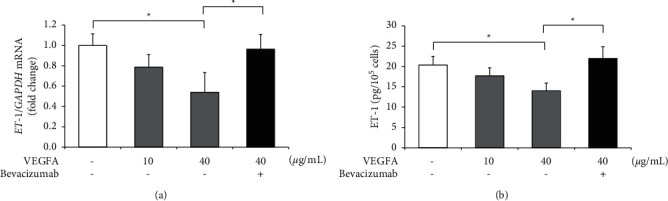
Vascular endothelial growth factor A (VEGFA) is associated with the suppression of ET-1 production in cultured human glomerular microvascular endothelial cells (hGECs). hGECs were plated in 24-well plates (1 × 10^5^ cells/well) for ELISA or 60 mm culture dishes (1 × 10^6^ cells/dish) for RT-PCR. hGECs that were 90% confluent were serum-starved for 24 h before the experiments were performed. hGECs were treated with 0, 10, and 40 ng/mL of VEGFA or with a combination of 40 ng/mL VEGFA and 1 *µ*M bevacizumab for 8 h. The levels of *ET-1* mRNA (a) and ET-1 protein (b) were measured using real-time RT-PCR and the ELISA after VEGFA and bevacizumab treatment. Data shown are means ± SD (*n* = 3; ^*∗*^*p* < 0.05). The experiments were repeated at least three times, with reproducible results.

To gain further insight into the relationship between bevacizumab and its target VEGFA, we determined the effect of VEGFA on ET-1 production in hGECs. We treated dose-adjusted VEGFA with hGECs in a serum-free medium without any growth factors. The mRNA and protein levels of ET-1 were markedly downregulated in VEGFA-treated hGECs (Figures 4(a) and 4(b)). In addition, the mRNA and protein levels of ET-1 returned to normal after bevacizumab treatment (Figures 4(a) and 4(b)).

## 4. Discussion

**Figure 5:**
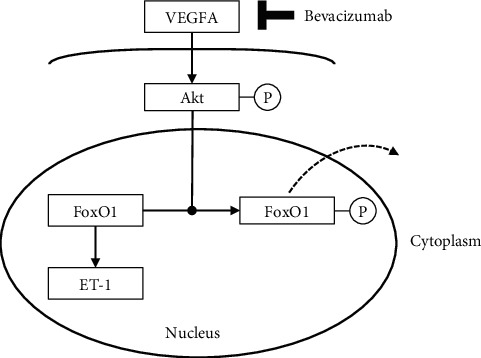
A schematic showing signaling pathways that increase ET-1 production on FoxO1 nuclear localization induced by bevacizumab in human glomerular microvascular endothelial cells (hGECs). Abbreviations: VEGFA, vascular endothelial growth factor A; Akt, protein kinase B; FoxO1, forkhead box protein O1; ET-1, endothelin-1.

In this study, we aimed to evaluate the effect of bevacizumab on ET-1 production in human glomerular microvascular endothelial cells (hGECs). We found that bevacizumab increased ET-1 production in cultured hGECs and the “Akt/FoxO1” pathway could be involved in the increased ET-1 production induced by bevacizumab (Figure 5). Importantly, ET-1 is involved in podocyte injury and proteinuria, and glomerular injury caused by increased ET-1 production may be more pronounced in the development and progression of bevacizumab-induced nephrotoxicity [22, 23]. Furthermore, we elucidated an intracellular mechanism that involves increased ET-1 levels during Akt inactivation and FoxO1 nuclear localization. Thus, this study indicated that ET-1 axis activation, Akt inactivation, and FoxO1 nuclear localization are the intracellular molecular mechanisms underlying bevacizumab-induced nephrotoxicity.

A previous in vitro study using human lung microvascular endothelial cells showed that VEGFA may maintain vascular homeostasis by decreasing ET-1 levels and the blockade of VEGF signaling by SU5416 increases ET-1 levels [24]. Although these results may seem obvious, we found that VEGFA decreased ET-1 production in a dose-dependent manner in the glomerular endothelium. Thus, our data support the potential role of VEGFA in the negative regulation of the ET-1 axis in the glomerular endothelium. However, the function of VEGFA in normal vascular endothelium is unknown. In contrast with our results, other studies indicated that VEGFA stimulates the synthesis and secretion of ET-1 and directly enhances the ET-1 axis in endothelial cells [25]. We believe that there are several reasons for this discrepancy. The main reason is that the source and type of endothelial cells used in our study and previous studies are different. Endothelial cells from different sources and types have different morphologies, structures, and functions [26]. To provide insights into the mechanism by which VEGFA regulates ET-1 in human glomerular endothelium, clarifying how VEGFA alters its downstream targets is necessary.

Our findings support the results of previous in vivo studies showing that treatment with multitarget VEGFR inhibitors is linked to increased ET-1 levels. Previous studies have reported that sunitinib administration is accompanied by increased blood pressure, renal injury, proteinuria, and circulating ET-1 levels [15]. Moreover, the increase in blood pressure and proteinuria could be prevented by the dual ETA/B receptor antagonist macitentan, indicating that ET-1 axis activation is critical for developing these side effects [15]. The role of ET-1 in regulating blood pressure and renal hemodynamics is well established [22]. According to the fundamentals of renal biology, the effect of the renal ET-1 system could be determined within the local microenvironment since renal-derived ET-1 primarily acts in an autocrine or paracrine manner [27, 28]. The kidney contains abundant ET receptors, especially in the vascular system, such as glomerular capillaries, and exhibits greater sensitivity to the vascular effects of ET-1 than other organs [28], In addition, glomerular endothelial cells are the principal source of ET-1 in the kidneys. In a study of the renal biopsies of patients with glomerular disorders, the severity of proteinuria was associated with the *ET-1* mRNA level in the glomeruli [29]. Thus, the renal ET-1 system activation is observed in various kidney disorders, including diabetic nephropathy, glomerulonephritis, and preeclampsia, suggesting that it can directly trigger proteinuria [22, 23].

FoxO1 is the predominant FoxO isoform expressed in the vascular endothelium [30]. Previous studies have reported that ET-1 production by endothelial cells is regulated via the PI3k/Akt/FoxO1 signaling pathway [19, 31]. This is related to enhanced *ET-1* promoter activity and FoxO1 nuclear localization. The VEGF-deficient endothelium showed increased FoxO1 protein levels in the nucleus and cytoplasm in vitro and in vivo [32]. In our study, FoxO1 protein levels in the nucleus of bevacizumab-treated hGECs increased. Moreover, we found that the Akt pathway was blocked by bevacizumab, and decreased Akt activity was associated with increased ET-1 production. Thus, one mechanism for the bevacizumab-induced increase in ET-1 production is likely the downregulation of Akt-induced FoxO1 phosphorylation, resulting in increased nuclear FoxO1 levels, which promotes the activity of the *ET-1* promoter. In addition, the AS1842856 inhibitor decreased ET-1 levels, suggesting that FoxO1 inhibition could alleviate the bevacizumab-induced increase in ET-1 production. Therefore, FoxO1 might be considered a potential drug target for the treatment of bevacizumab-induced nephrotoxicity. However, whether bevacizumab promotes ET-1 and nuclear FoxO1 levels by directly inhibiting the Akt pathway remains unclear and requires further studies.

Collectively, VEGFA depletion by bevacizumab increased ET-1 levels in hGECs. Thus, the inhibition of renal ET-1 production could be a promising approach to protect against or treat bevacizumab-induced nephrotoxicity considering the known detrimental effects of ET-1 on the kidney. Moreover, our findings provide novel insights into “Akt/FoxO1” in increased ET-1 production by bevacizumab treatment.

## Data Availability

All data analyzed during this study are included within this manuscript.
